# Correction to “Efficacy of Trametinib in a Metastatic Urothelial Carcinoma Patient With a BRAF Mutation: A Case Report”

**DOI:** 10.1002/iju5.70047

**Published:** 2025-06-18

H. Karasawa, Y. Yasumizu, T. Kosaka, Shimoi, and M. Oya, “Efficacy of trametinib in a metastatic urothelial carcinoma patientwith a BRAF mutation,” *IJU Case Reports* 7, no. 5 (2024): 375‐378, https://doi.org/10.1002/iju5.12759.

In the ‘Case presentation’ section, the first two sentences in the second paragraph read as follows: To provide further treatment for this patient, we conducted cancer multigene panel testing (FoundationOne®). We examined 324 cancer‐related genes and identified the BRAF G469A mutations (Fig. 2).

The authors would like to clarify that the cancer panel test was performed at the referring hospital and not at the authors’ hospital. The correct text should be: To provide further treatment for this patient, cancer multigene panel testing (FoundationOne®) was conducted. Analysis of 324 cancer‐related genes revealed the BRAF G469A mutation (Fig. 2).

